# Symptom Networks in Patients Receiving Dialysis: A Systematic Review of Node Centrality, Bridge Centrality, and Methodological Quality

**DOI:** 10.3390/healthcare14213583

**Published:** 2026-10-23

**Authors:** Yiran Chen, Jieling Chen, Jing Wang, Jiaxin Liu

**Affiliations:** 1 School of Nursing, Sun Yat-sen University, 74 Zhongshan Er Road, Yuexiu District, Guangzhou 510080, China; chenyr77@mail2.sysu.edu.cn (Y.C.); chenjling23@mail.sysu.edu.cn (J.C.); wangj788@mail2.sysu.edu.cn (J.W.); 2 School of Nursing, Guangdong Medical University, Xincheng Blvd, Songshan Lake National High-Tech Industrial Development Zone, Dongguan 523808, China

**Keywords:** dialysis, symptom network, network analysis, centrality, bridge symptoms, methodological quality, systematic review

## Abstract

**Highlights:**

**What are the main findings?**

- In a descriptive synthesis of nine networks, anxiety, difficulty falling asleep, and sadness ranked highest for node strength.
- Fatigue was the most recurrent connecting symptom, while methodological quality was mostly low to moderate and all available evidence was cross-sectional.

**What are the implications of the main findings?**

- Dialysis symptom assessment should integrate psychological, sleep, and fatigue-related domains rather than focus only on isolated physical symptoms.
- Longitudinal, covariate-adjusted, and transparently reported network studies are needed before central symptoms are treated as causal intervention targets.

**Abstract:**

Background/Objectives: Patients receiving dialysis experience multiple interacting physical and psychological symptoms. Network analysis can identify symptoms occupying central or bridging positions, but findings across dialysis studies have not been systematically synthesized. This review aimed to synthesize central and bridge symptoms in adults receiving dialysis and evaluate the methodological quality of symptom network studies. Methods: Following PRISMA 2020 and a prospectively registered protocol (PROSPERO CRD420261410561), PubMed, Scopus, Web of Science, CINAHL, China National Knowledge Infrastructure, and Wanfang Data were searched from inception to 9 May 2026. Two reviewers independently screened studies and assessed methodological quality. Centrality rankings from studies using the Dialysis Symptom Index or modified versions were min-max normalized and synthesized. Results: Sixteen cross-sectional studies involving 7691 participants were included; 15 were conducted in mainland China and one in Singapore, while 14 involved hemodialysis populations and two involved peritoneal dialysis populations. Nine network models contributed to the centrality-ranking synthesis. Anxiety ranked first (median normalized rank 0.0690), followed by difficulty falling asleep (0.2353) and sadness (0.2400). Fatigue and irritability were most frequently identified as bridge or connecting symptoms. Thirteen studies reported strength correlation-stability coefficients of at least 0.50. Seven studies were rated low quality, eight moderate quality, and one high quality, with recurrent deficiencies in model-selection reporting, subgroup comparisons, raw centrality values, and uncertainty assessment. Conclusions: Psychological and sleep-related symptoms occupied prominent positions in the analyzed networks, while fatigue frequently connected symptom communities. The evidence predominantly reflects hemodialysis samples from mainland China. The applicability to peritoneal dialysis populations and to other healthcare systems and cultural settings remains uncertain. These cross-sectional findings are hypothesis-generating and require validation in more diverse populations.

## 1. Introduction

Although dialysis can replace some kidney functions, current medical technology cannot fully correct the uremic milieu. Patients with end-stage kidney disease (ESKD) receiving dialysis therefore continue to experience multiple symptoms, including fatigue, lack of appetite, and pruritus [1]. These symptoms do not occur in isolation; instead, they may interact with one another and adversely affect physical functioning, quality of life, and clinical outcomes. Previous studies have described symptom burden, patterns of symptom associations [2,3], and associated factors [4,5]. However, prevalence and severity estimates, dimension-reduction methods, and clustering analyses, although useful for describing symptom distribution, do not directly characterize the network of conditional relationships among symptoms or identify symptoms that are especially connected within that network. More refined analytical approaches are therefore needed [6].

With advances in information technology, big-data analytics, and methodology, symptom network analysis has emerged as an approach for investigating relationships among symptoms [7]. In a symptom network, symptoms are represented as nodes and conditional dependencies between symptoms as edges. This framework visualizes the pattern and strength of symptom associations. Centrality indices, such as node strength and expected influence, quantify how strongly a symptom is connected with other symptoms and can help identify candidate symptoms for prioritized assessment and further investigation [6]. In a symptom network of 354 patients receiving maintenance hemodialysis, Chang et al. found that worry had the highest node strength, whereas fatigue had the highest bridge strength and connected psychological and uremia-related symptom communities [8]. Such findings can generate clinically relevant hypotheses, but cross-sectional centrality alone does not establish that intervening on a central or bridge symptom will improve other symptoms.

The application of symptom network analysis to dialysis-related symptoms is still an emerging field. To our knowledge, no systematic review has yet synthesized the available evidence on symptom networks in patients receiving dialysis. Existing studies are fragmented, their populations differ in socio-demographic and clinical characteristics, and their choices of measurement tools, node definitions, network estimation models, and stability assessments vary. As a result, it remains unclear which symptoms consistently emerge as central or bridging across studies, and whether these findings are robust enough to inform symptom management.

Accordingly, this systematic review aimed to synthesize the application of symptom network analysis in adults receiving dialysis, identify symptoms that repeatedly occupy central or bridge positions, and appraise the methodological quality of the available evidence. Specifically, the objectives were to: (1) describe the characteristics, dialysis modalities, symptom measures, and principal findings of existing studies; (2) summarize network estimation methods, centrality and bridge-centrality indices, and accuracy and stability assessments; and (3) synthesize centrality rankings across studies using comparable symptom measures. The synthesis was intended to clarify clinically relevant symptom patterns while distinguishing associative network findings from causal or temporal effects.

## 2. Materials and Methods

### 2.1. Design and Registration

A systematic review of published symptom network studies in adults receiving dialysis was undertaken. The review was reported in accordance with the Preferred Reporting Items for Systematic Reviews and Meta-Analyses 2020 statement [9,10] and was prospectively registered in the International Prospective Register of Systematic Reviews (registration number: CRD420261410561; https://www.crd.york.ac.uk/PROSPERO/view/CRD420261410561; accessed on 18 October 2026).

### 2.2. Search Strategy

PubMed, Scopus, Web of Science, CINAHL, China National Knowledge Infrastructure (CNKI), and Wanfang Data were searched from database inception to 9 May 2026. No language restrictions were applied during the database searches. However, only studies published in English or Chinese were eligible for inclusion. Search terms included dialys*, hemodialys*, peritoneal dialys*, ESKD, ESRD, symptom, and network analysis. The database-specific search strings and Boolean combinations are provided in Supplementary Material S1. To identify potentially eligible publications beyond the database searches, the reference lists of all included studies were manually screened through backward citation tracking. No additional eligible studies were identified through this manual search.

### 2.3. Eligibility Criteria

Eligibility criteria were developed using the PICOS framework to define the scope of study selection. The inclusion criteria were as follows: (1) Population (P): adults receiving hemodialysis or peritoneal dialysis; (2) Intervention (I): not applicable; (3) Comparator (C): not applicable; (4) Outcomes (O): physical or psychological symptoms, symptom burden, symptom clusters, or closely related psychosocial and cognitive manifestations, such as depression, stigma, fatigue, and cognitive complaints, among patients receiving dialysis; and (5) Study design (S): use of a network analytic method, including but not limited to correlation networks, partial correlation networks, Gaussian graphical models, EBICglasso, LASSO, mixed graphical models, bridge network analysis, or other psychometric network models. Studies were also required to report at least one network result, such as network structure, edge weights, centrality, bridge centrality, predictability, or network stability.

### 2.4. Study Selection, Data Extraction, and Synthesis

Retrieved records were uploaded to Rayyan, a web application for systematic reviews [11]. After duplicate removal, two reviewers independently screened records and assessed full texts; disagreements were resolved by a third reviewer. One reviewer independently extracted the data. Following previous reviews [12,13], when a study constructed multiple network models from different cross-sectional assessments, all such models were included. When more than one network model was estimated from the same dataset, only the model with the largest sample was included in the analysis. When multidimensional symptom networks were reported, only the overall symptom network was included. Extracted data comprised first author, country, study population, sample size, age, symptom assessment tool, network method, and main findings. A second reviewer checked all extracted data.

Network findings were summarized according to the metrics reported. Node strength represents the sum of the absolute weights of edges connected to a node, whereas one-step expected influence retains the signs of those weights [14,15]. Betweenness describes a node’s position on shortest paths between other nodes, while bridge strength summarizes the absolute weights of connections to nodes outside its assigned symptom community [16,17]. These measures were not treated as interchangeable. The descriptive ranking synthesis combined node-strength rankings only; expected influence, betweenness, closeness, bridge-centrality measures, and predictability were considered separately.

A rank-based synthesis was used because complete numerical strength estimates and their uncertainty were not consistently available across studies. Previous reviews have also summarized centrality using within-network rankings [13,18]. Symptom labels were harmonized according to their item content, and the number of symptom nodes actually included in each selected network was recorded. Within-network strength ranks were min-max normalized as follows:

$$N=\frac{X-X_{\min}}{X_{\max}-X_{\min}}$$

where *N* is the normalized rank, *X* is a symptom’s strength rank within a network, and *X*_min and *X*_max are the smallest and largest ranks in that network, respectively. Rank 1 indicates the highest strength. This transformation places ranks on a common 0–1 scale while preserving their within-network ordering; lower values indicate higher relative strength ranks. It permits a descriptive comparison of relative positions across networks with different numerical ranges of ranks, but does not establish equivalence between networks or imply that identical normalized ranks represent equal absolute strength.

For each symptom, normalized ranks were summarized using the median and interquartile range, with each contributing study given equal weight. These summaries describe the distribution of relative ranks across studies and are not precision-weighted pooled centrality estimates. Symptoms absent from a network were treated as missing and were not assigned zero or the lowest rank. Symptom-specific study contributions were determined from non-missing entries and are reported in Supplementary Table S1. Where published figures did not allow a unique rank to be established, the plausible rank range was retained, and its lower and upper bounds were normalized and summarized separately. The resulting bounds reflect extraction uncertainty rather than confidence intervals or uncertainty in the original network estimates.

Following general guidance on examining the sensitivity of systematic-review findings to analytical and study-selection decisions, including syntheses without effect-size meta-analysis, two complementary types of post hoc sensitivity analysis were conducted [19,20].

First, restricted syntheses examined whether the inclusion of networks with reduced node sets affected the descriptive rankings. One analysis excluded Zhai et al. [21] and L. Zhang, Han, et al. [22], whose networks contained 18 and 22 symptoms, respectively, leaving seven studies. These were the two smallest node sets among the nine included networks. A further restriction retained only the five networks containing 30 symptom nodes, the largest and most frequently represented node count in the primary synthesis. These restrictions provided progressively narrower comparisons based on network size. They were exploratory decisions based on the characteristics of the included studies, not validated thresholds for network comparability.

Second, the study-deletion principle used in influence assessment was adapted to the descriptive ranking synthesis to examine dependence on individual studies [23]. Each of the nine studies was omitted in turn, and symptom-specific medians and interquartile ranges of normalized ranks were recalculated using the remaining eight studies. All sensitivity analyses retained the same symptom mappings, within-network normalization, missing-value handling, equal study weights, and treatment of extraction uncertainty as the primary analysis. Symptom-specific study counts were also recalculated. These analyses examined changes in descriptive summaries of published rankings; they did not re-estimate the original networks using a common node set.

### 2.5. Methodological Quality Assessment

Because network analysis is a relatively new method, no universally accepted guideline is available for evaluating network-model quality. A Dutch research team developed a quality assessment tool for cross-sectional network analyses [24] based on the 2023 reporting standards for symptom network analyses proposed by Burger et al. [25]. The tool contains 18 items across three domains: sample and reporting quality, analytic quality, and open science practices. Within each domain, studies scoring <50% of the maximum were classified as low quality, those scoring 50–74% as moderate quality, and those scoring ≥75% as high quality. Two reviewers independently assessed each included study. Disagreements were resolved through discussion and, when necessary, adjudication by a third reviewer.

## 3. Results

### 3.1. Search Results

**Figure 1:**
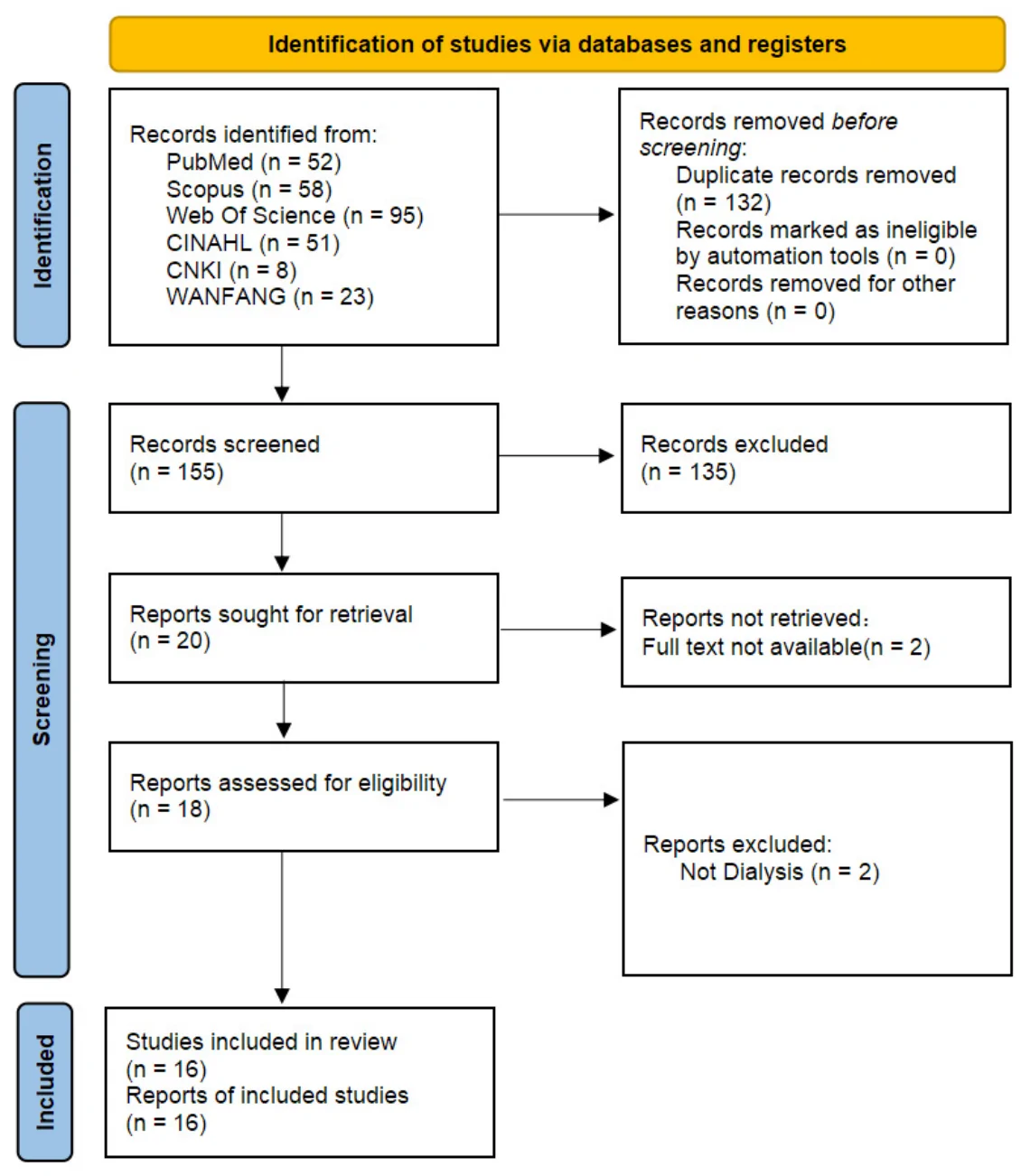
PRISMA flow diagram of study selection.

The database search yielded 287 records. After deduplication, title and abstract screening, and full-text review, 16 studies were included: eight published in Chinese [4,21,22,26,27,28,29,30] and eight published in English [8,31,32,33,34,35,36,37]. The study selection process is shown in Figure 1.

### 3.2. Overview of Included Studies

Characteristics of the included studies are presented in Table 1. All 16 studies used cross-sectional designs and were published between 2020 and 2026. Fourteen studies included patients receiving hemodialysis, and two included patients receiving peritoneal dialysis. One study was conducted in Singapore; all others were conducted in mainland China. Sample sizes ranged from 147 to 1866, with a total of 7691 patients. Except for studies that did not report age or reported age as a median and interquartile range, mean participant age generally ranged from 47.80 to 70.05 years. No fully or partially overlapping participant samples were identified across the included publications; therefore, no participants were counted more than once across publications in this review. One study specifically enrolled older adults receiving maintenance hemodialysis. The proportion of women ranged from 34.3% to 52.66%.

### 3.3. Symptom Assessment Tools

Based on the constructs assessed, the instruments were classified as kidney disease–specific symptom measures, psychosocial functioning measures, and cognitive functioning measures. Kidney disease–specific instruments were used most frequently. Eleven studies used the Dialysis Symptom Index (DSI) or a modified version to assess symptom frequency, severity, or distress [4,8,21,22,26,28,30,34,35,36,37]. Other instruments were each used once, including the Post-Dialysis Fatigue Scale (PDF) [29], Dialysis Frequency, Severity, and Symptom Burden Index (DFSSBI) [27], Chronic Kidney Disease Symptom Burden Index (CKD-SBI) [32], and Kidney Disease Questionnaire (KDQ) [8]. Psychosocial functioning measures assessed depressive symptoms and the illness-related social impact of dialysis, including the Patient Health Questionnaire-9 (PHQ-9; *n* = 1) [33] and the Social Impact Scale (SIS; *n* = 2) [33,34]. Cognitive functioning was assessed using the Patient Assessment of Own Functioning Inventory (PAOFI) and the Montreal Cognitive Assessment (MoCA) [31].

### 3.4. Network Analysis Approach

The included studies used several network estimation methods and models: regularized partial correlation networks (EBICglasso; *n* = 10) [8,26,27,28,29,30,31,33,34,35]; a pairwise Markov random field (*n* = 1) [8]; and an Ising model (estimateNetwork/NIRA; *n* = 1) [36]. Four studies did not clearly report the specific network estimation method [4,22,32,37]. All reported networks used the Fruchterman–Reingold algorithm to determine node placement and visualize network structure.

### 3.5. Methodological Quality

The quality assessment results are presented in Table 2. The 16 studies were evaluated across 18 items in three domains: sample and reporting quality, analytic quality, and openness. Total scores ranged from 7 to 13, with a mean of 9.25 ± 1.61. Seven studies were rated low quality, eight moderate quality, and one high quality. Sample and reporting quality was generally satisfactory, with most studies reporting demographic and clinical information, sample size, and data preprocessing. Analytic quality varied. The main limitations were incomplete reporting of model-selection criteria, subgroup analysis methods, raw centrality scores, edge-weight accuracy, and other uncertainty assessments.

### 3.6. Node Centrality

Strength was the most widely used centrality index: all 16 studies used strength to identify central symptoms. Reported central symptoms included psychological symptoms (e.g., sadness, irritability, and anxiety) [8,21,22,26,27,30,34], fatigue or lack of energy [32,36], sleep disturbance [4,37], and pain-related symptoms [34,35].

### 3.7. Symptom Centrality Rankings

Nine studies with symptom items that could be harmonized and extractable node-strength rankings contributed to the descriptive ranking synthesis. Each study contributed one network containing 18–30 symptom nodes. Chang et al. [8], which retained only 13 of the 30 DSI symptoms after symptom selection, was excluded from this synthesis because of its markedly restricted node set. Cheng et al. [4] were excluded because rankings for all symptoms in the selected network could not be extracted. These studies remained included in the systematic review and narrative synthesis.

**Figure 2:**
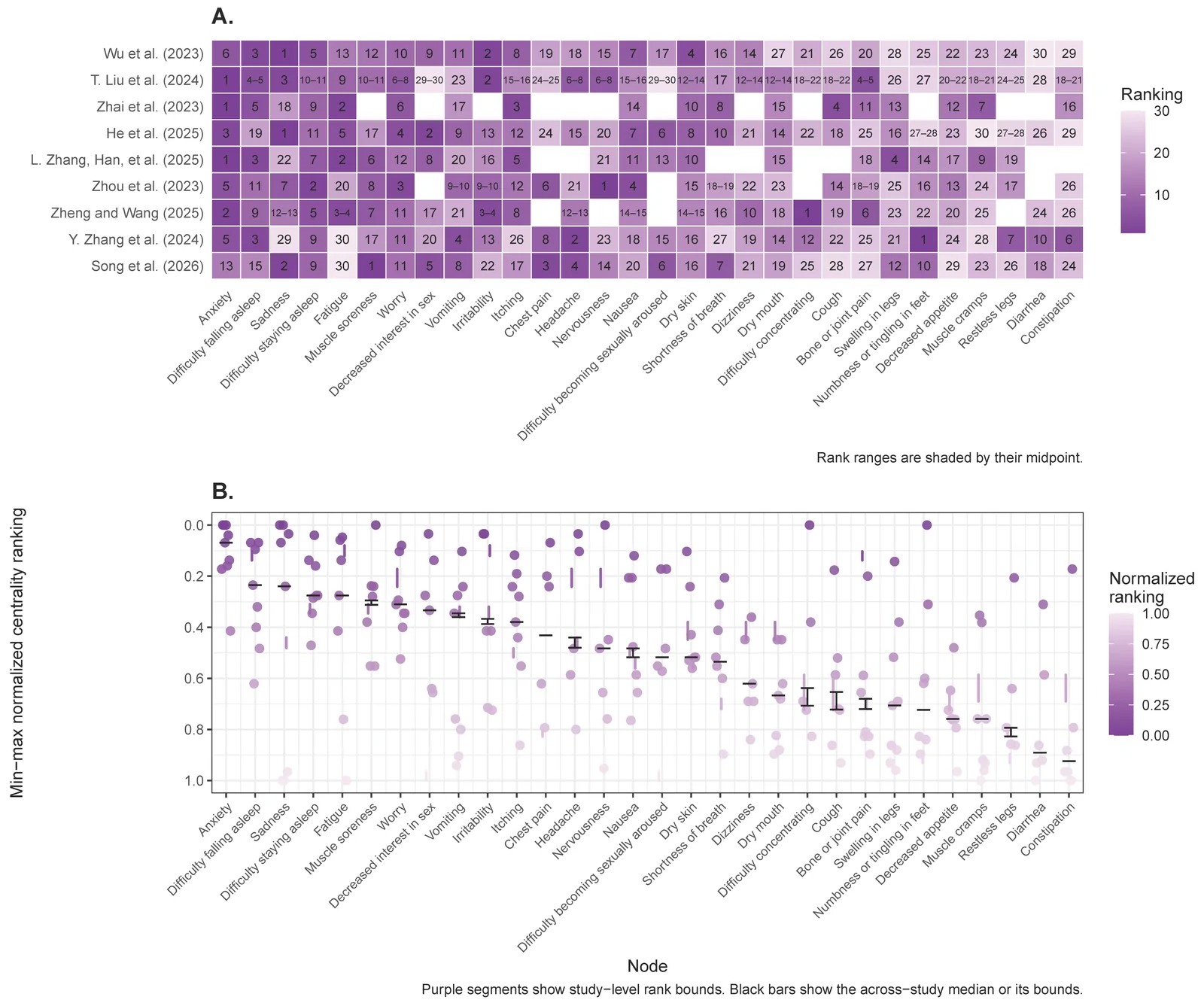
Centrality rankings of symptom network models in patients receiving dialysis. Data were extracted from nine studies [21,22,26,27,30,34,35,36,37]. (**A**) Symptom strength centrality rankings across studies. (**B**) Distribution of min–max normalized symptom strength centrality rankings.

Across the nine networks, 30 distinct symptom labels were represented, with contributions ranging from six to nine studies per symptom. Supplementary Table S1 provides symptom-specific study counts, contributing studies, and extracted ranking information. Anxiety, difficulty falling asleep, and sadness each received contributions from all nine studies and had the three lowest median normalized strength ranks. Their median normalized ranks were 0.0690 (IQR, 0.0000–0.1600), 0.2353 (IQR, 0.0952–0.4000), and 0.2400 (IQR, 0.0345–0.9655), respectively. The symptom centrality rankings across the nine networks are presented in Figure 2. 

In the seven-study restricted synthesis, the median normalized ranks were 0.1379 for anxiety, 0.3200 for difficulty falling asleep, and 0.0690 for sadness. In the synthesis restricted to five 30-node networks, the corresponding values were 0.1379, 0.1034–0.1379, and 0.0345. The range for difficulty falling asleep reflects extraction uncertainty. Sadness had the highest relative strength ranking in both restricted syntheses, indicating that the ordering observed in the primary analysis changed with study selection. Both restricted subsets contained only hemodialysis studies. Full symptom-specific results are presented in Supplementary Table S2.

Across the nine leave-one-study-out analyses, anxiety retained the highest relative strength ranking, with median normalized ranks ranging from 0.0545 to 0.1034. The relative positions of difficulty falling asleep and sadness varied across omissions. These findings indicate that anxiety’s leading position was maintained after any single-study omission, although the complete symptom ordering was sensitive to changes in the contributing studies. Results are presented in Supplementary Table S3.

### 3.8. Bridge Symptoms

Bridge-strength findings were summarized separately from findings based on betweenness centrality. Among studies reporting bridge strength, fatigue was identified as a bridge symptom in two studies [8,21]. Other reported bridge symptoms included irritability [30], worry [28], anxiety [27], and difficulty falling asleep [4].

Several studies also used betweenness centrality to describe potential connecting symptoms [26,27,35,37]. These findings concern shortest-path positions within the overall network and were not counted as additional bridge-strength findings. Comparisons of bridge-strength results also require consideration of how each study defined symptom communities.

### 3.9. Accuracy and Stability of Network Indices

Eight studies used nonparametric bootstrapping to estimate 95% confidence intervals (CIs) for edge weights [4,8,21,33,34,37]. One reported that CI widths ranged from narrow to moderate [32], and one merely stated that 95% CIs were used to assess edge-weight accuracy without reporting the numerical estimates [29]. Three studies performed bootstrap difference tests [8,29,32]. Two examined differences between edge weights [8,32], while all three examined differences in centrality indices, including node strength or bridge centrality. Only Chang et al. [8] reported the specific results of the edge-weight difference test.

All 16 studies reported correlation-stability (CS) coefficients for strength centrality; 13 reported values of ≥0.50, while the remaining three reported values from 0.25 to <0.50, indicating acceptable stability across all studies.

## 4. Discussion

This is the first systematic review to comprehensively evaluate central symptoms in patients receiving dialysis on the basis of network analyses, and it included 16 studies. Two main findings emerged. First, in the descriptive synthesis of nine networks, anxiety, difficulty falling asleep, and sadness had the highest overall node-strength rankings. Fatigue and irritability were the symptoms most frequently identified as bridges. Second, the overall methodological quality of the included studies was low to moderate: seven studies were rated low quality, eight moderate quality, and only one high quality. Thus, this review identified key symptoms in dialysis symptom networks while also revealing deficiencies in methodological standardization and completeness of reporting.

### 4.1. Central Symptoms

In the primary nine-network synthesis, anxiety had the highest relative strength ranking. The associations reported in individual studies provide context for this finding. Anxiety was strongly associated with worry in hemodialysis studies and in a peritoneal dialysis study [8,21,22], while Wu et al. [30] reported a relatively strong connection between anxiety and irritability. These findings locate anxiety within a group of interconnected emotional symptoms. Outside the network literature, Kose and Mohamed [38] also found a positive association between anxiety and depressive symptom scores in patients receiving hemodialysis. Taken together, these observations suggest that anxiety’s prominent network position may partly reflect its connections with other manifestations of emotional distress. Further research could examine whether these connections persist when shared psychosocial factors and overlap in symptom content are considered.

Difficulty falling asleep was also prominent in the primary ranking synthesis. A recurring finding across the included studies was its strong association with difficulty staying asleep, reported in both hemodialysis and peritoneal dialysis samples [21,22,27,30,37]. These repeated associations suggest that difficulties initiating and maintaining sleep are closely related components of the symptom experience in these samples. External evidence extends this discussion to emotional symptoms: Kose and Mohamed [38] found that higher sleep-latency scores were associated with greater anxiety and depressive symptoms. Temporal evidence presents a more differentiated picture. In a three-month longitudinal study, Tan et al. [39] found that baseline fatigue and depressive symptoms were associated with subsequent overall sleep quality, whereas baseline sleep quality did not significantly predict subsequent fatigue or depressive symptoms in the overall sample. These findings provide a rationale for investigating the temporal relationships among sleep, fatigue, and emotional symptoms without assuming a uniform direction of association.

Sadness’s prominence in the primary ranking synthesis was accompanied by reported connections with other emotional symptoms. Wu et al. [30] and He et al. [26] identified relatively strong associations between sadness and worry, while Zheng and Wang [36] described strong interrelationships among worry, sadness, and anxiety. Qualitative evidence offers a possible psychosocial context for these findings. In interviews with older adults receiving hemodialysis or peritoneal dialysis, Raj et al. [40] identified experiences of lost autonomy, disruption of previous ways of living, and uncertainty about symptoms and the future. Such experiences may provide a shared context for sadness, worry, and anxiety to occur together. This interpretation supports examining their common psychosocial correlates, while recognizing that a single sadness item does not establish a diagnosis of depressive disorder.

Overall, the rankings and reported symptom associations identify emotional and sleep-related patterns for further investigation. They generate hypotheses about symptom interrelationships; establishing temporal ordering and determining whether modifying a particular symptom improves other symptoms will require longitudinal and intervention studies.

### 4.2. Bridge Symptoms

Fatigue had the highest bridge strength in two included studies of patients receiving maintenance hemodialysis [8,21]. Zhai et al. [21] assigned fatigue to the general uremic symptom community and described its connections with emotional and sleep-related symptom communities. These findings suggest that fatigue may occupy a connecting position across symptom domains in the hemodialysis samples studied. Findings based on betweenness centrality were interpreted separately, as this metric describes a symptom’s position on shortest paths within the overall network.

Evidence from studies outside the network literature provides context for these associations. Bossola et al. [41] found that patients reporting fatigue more frequently experienced itching, muscle soreness, bone or joint pain, sadness, and anxiety, and reported more severe difficulty staying asleep. In the baseline analysis of the CHOICE cohort, which included patients receiving hemodialysis and peritoneal dialysis, lower vitality scores, indicating greater fatigue, were associated with poorer mental health, greater bodily pain, and poorer sleep quality [42]. These observations suggest that fatigue accompanies several dimensions of symptom burden. Shared clinical and psychosocial factors may contribute to this pattern, providing a possible explanation for fatigue’s connections across symptom communities.

Bridge-strength findings also varied across the included studies. Other identified bridge symptoms included irritability [30], worry [28], anxiety [27], and difficulty falling asleep [4]. Differences in symptom-community definitions are relevant to this variation. For example, Zhai et al. [21] placed fatigue and sleep difficulties in separate communities, whereas Ma et al. [28] grouped fatigue, difficulty falling asleep, and difficulty staying asleep within the same uremic symptom community. Whether a fatigue–sleep connection contributes to bridge strength consequently depends on the community assignment. Bridge status should therefore be interpreted in relation to both the estimated connections and the way symptoms were grouped. Future studies should report the specific cross-community connections underlying bridge findings and examine their reproducibility using comparable symptom sets and clearly specified community definitions.

### 4.3. Methodological Issues

Basic study information was generally reported adequately: most studies described demographic and clinical characteristics, sample size, network model, analytic software, and centrality findings. However, closer evaluation of network analytic quality revealed substantial reporting deficiencies in model-selection criteria, details of subgroup analyses, raw centrality results, and uncertainty assessments.

First, model-selection criteria were insufficiently reported. Reporting standards for psychological network analyses recommend that cross-sectional network studies clearly describe the network estimation method, type of correlation or covariance matrix, model-selection procedure, software package, and key parameter settings to limit researcher degrees of freedom and improve reproducibility [25]. Only two included studies explicitly reported model-selection criteria [31,33]. Inadequate reporting of model-selection procedures and parameter settings increases analytic flexibility during network construction and prevents readers from determining whether between-study differences reflect genuine symptom structures or different models and settings. Future studies should fully report the network estimation method, rationale for model selection, key parameters, and software versions to improve transparency and reproducibility.

Second, very few dialysis symptom network studies performed subgroup comparisons. Network analyses in other disease areas suggest that network structures may differ by treatment stage, symptom burden, and clinical characteristics [13,43,44]. In the systematic review of depressive symptom networks by Malgaroli et al. [13], 10 studies compared 31 networks across groups. Some reported differences by sex, country, disease status, or treatment stage, whereas no significant differences were found between subgroups defined by anxiety level, depressive symptom severity, treatment response, or genetic or environmental risk. Subgroup comparisons assess whether an overall network obscures heterogeneity and whether central symptoms and symptom associations generalize across patient groups. Detected differences can generate hypotheses for stratified symptom management; null findings can support a degree of cross-group consistency. Although subgroup analysis is not mandatory in every network study, it has substantial methodological value when precision symptom management is the aim and dialysis modality, treatment stage, or clinical characteristics may influence symptom presentation.

Third, reporting of raw centrality results was incomplete. Although most studies identified central symptoms or displayed rankings in standardized centrality plots, none reported raw values for all centrality indices calculated. Burger et al. [25] recommended reporting every calculated centrality index and providing raw centrality values in a supplementary table or appendix; exact parameter values cannot be recovered reliably from plots alone, and presentation as Z scores may exaggerate differences between nodes. Missing raw values therefore make it difficult to verify the identification and ranking of central symptoms and limit the accurate extraction and validation of centrality findings in this review. Future studies should provide tabulated raw values for every centrality index at each node, together with standardized results and rankings where useful. For bridge centrality, the basis for defining symptom communities and the corresponding raw values should also be reported to improve transparency and usability.

Fourth, uncertainty assessment was incomplete in many studies. Epskamp et al. [14] recommended using bootstrap procedures to assess edge-weight accuracy, centrality stability, and differences between edge weights or centrality estimates, and using the CS coefficient to evaluate the stability of centrality under case-dropping perturbation. Most dialysis symptom network studies reported CS coefficients, indicating awareness of the importance of stability testing when interpreting central symptoms. However, some did not report edge-weight accuracy, centrality difference tests, or bootstrap confidence intervals, and did not clearly state the number of bootstrap or subsampling iterations. Although edge weights were not the primary outcome of this review, edge-weight accuracy remains important for judging the reliability of the estimated network structure and provides necessary context for interpreting centrality and bridge-centrality findings.

### 4.4. Strengths and Limitations

This review and the available evidence also have limitations. First, all included studies used cross-sectional designs. Their networks describe associations at a single assessment and do not establish temporal ordering, causality, or treatment responsiveness. High node or bridge centrality does not demonstrate that a symptom initiates other symptoms or that treating it will reduce overall symptom burden [16,45]. Model-based simulations, such as those reported by Zheng and Wang [36], generate predictions under the fitted model and do not constitute observed clinical treatment effects. The identified central and bridge symptoms should therefore be considered candidates for hypothesis-driven research.

Second, covariate handling was inadequate in existing dialysis symptom network studies. Sex, age, educational attainment, dialysis vintage, and comorbidities may affect both symptom presentation and intersymptom associations. Most studies kept these factors outside the network model and examined their associations with symptom burden, symptom clusters, or centrality indices only after network estimation through stratified, univariable, or multivariable regression analyses [4]. Such analyses can identify factors associated with symptom outcomes but cannot fully control confounding of network edges by shared disease course, treatment factors, and patient characteristics. Incorporating key covariates during network estimation may therefore improve the accuracy of estimated intersymptom associations.

Third, the ranking synthesis is conditional on the comparability of symptom content and the reliability of the extracted within-network ordering. Min-max normalization standardizes the numerical range of ranks but does not remove differences in symptom selection, prevalence thresholds, measurement dimensions, or network estimation. Rank transformation also discards the magnitude of differences between the original strength estimates: adjacent ranks may represent either small or substantial differences in strength. Moreover, each symptom’s summary depends on which studies included that symptom, and exclusion of low-prevalence symptoms may produce systematic differences in data coverage. Retaining extraction bounds addresses ambiguity in reading published figures but does not quantify uncertainty in the underlying network estimates.

The sensitivity analyses illustrate these limitations. Anxiety remained highest ranked after each individual study omission, whereas sadness ranked highest in both restricted syntheses. Thus, stability under single-study omission did not imply an invariant ordering across different study subsets. The restrictions were post hoc and exploratory, and changed both network composition and the populations represented; notably, both restricted subsets contained only hemodialysis studies. Differences between analyses therefore cannot be attributed solely to node selection. Furthermore, retaining networks with the same number of nodes does not ensure identical item content, measurement properties, or estimation methods. The resulting rankings should be interpreted as exploratory descriptions of relative positions within the analyzed networks, rather than a definitive hierarchy of symptom importance.

### 4.5. Clinical and Nursing Implications

This review identifies associations involving emotional symptoms, sleep difficulties, and fatigue that merit further investigation in the populations studied. For nursing research, a relevant question is whether repeated assessment of these domains helps characterize changes in patients’ overall symptom burden and daily functioning.

Clinical practice considerations draw on broader dialysis symptom-management literature. The KDIGO Controversies Conference report advocates combining open-ended questions about patients’ concerns with standardized patient-reported measures and prioritizing symptoms according to their impact on patients’ lives [1]. Within this approach, nurses can document emotional, sleep-related, and physical symptoms, discuss patients’ priorities, and coordinate individualized follow-up within the multidisciplinary team. Decisions about treatment and referral should be guided by clinical assessment, patient preferences, and evidence supporting the available interventions.

### 4.6. Directions for Future Research

Based on the findings and limitations of this review, future research can consider the following aspects: (1) Examine temporal relationships and evaluate intervention hypotheses. Longitudinal studies with repeated symptom assessments can examine symptom persistence and time-lagged associations. Randomized controlled trials, where feasible, are needed to test whether interventions addressing candidate symptoms improve those symptoms, other symptoms, and patient-reported functioning. Candidate symptoms should be selected using both network-generated hypotheses and an independent clinical rationale. (2) Control for covariates within the network. Sex, age, dialysis vintage, comorbidities, and other factors may confound intersymptom associations. Incorporating key covariates at the network estimation stage may better isolate direct symptom relationships and reduce confounding bias. (3) Improve methodological reporting. Researchers should follow network reporting standards and provide detailed descriptions of estimation methods and model-selection rationales, raw centrality values, edge weights and their 95% CIs, bootstrap difference tests, and stability coefficients. (4) Broaden population coverage and perform subgroup comparisons. Future studies should include more patients receiving peritoneal dialysis and recruit participants from a wider range of countries, healthcare systems, and cultural settings. Comparisons across dialysis modalities, settings, dialysis vintage, and symptom-burden levels could assess the reproducibility of symptom associations and centrality patterns and identify population-specific features.

## 5. Conclusions

This systematic review synthesized 16 cross-sectional symptom network studies, including 14 involving hemodialysis and two involving peritoneal dialysis; 15 were conducted in mainland China. In the descriptive synthesis of nine networks, anxiety, difficulty falling asleep, and sadness ranked highest for node strength. Fatigue was frequently reported as a connecting symptom in the included literature. Although both dialysis modalities were represented, the limited number of peritoneal dialysis studies precludes firm conclusions about the consistency of these patterns across modalities. The geographical concentration of the evidence also limits generalizability beyond mainland China. Methodological quality was predominantly low to moderate, with incomplete reporting of model-selection criteria, subgroup comparisons, raw centrality estimates, and uncertainty assessments. Given the cross-sectional designs, the predominance of studies from mainland China and hemodialysis populations, methodological heterogeneity, and reporting limitations, the findings should be interpreted as hypothesis-generating and do not establish causal intervention targets. Future research should prioritize multicenter longitudinal studies, clearer covariate handling, and standardized reporting to clarify temporal relationships and assess reproducibility. Intervention studies are also needed to determine whether targeting these symptoms improves patient outcomes.

## Figures and Tables

**Table 1:** Characteristics of the included studies.

First Author.	Study Period	Sociodemographic Information				Measurement Tool	Network Analysis						Results
		Sample Size	Age	Female n, (%)	Dialysis Modality		Correlation Analysis	Network Estimation	Model	Centrality Indices	CS Coefficient	Accuracy	
Wu et al. [30]	Dec 2022–Apr 2023	541	59.39 ± 13.62	194 (35.86)	MHD	DSI- Chinese	Not reported	Regularized Partial Correlation	EBIC- glasso	Strength Betweenness Closeness Bridge-Strength Predictability	0.75	Not Reported	Top three by strength: sadness, irritability, and difficulty falling asleep. Top three by bridge strength: irritability, sadness, and difficulty concentrating.
Tian et al. [29]	Jun–Sep 2021	147	Not Reported	59 (40.14)	MHD	PDF	Not reported	Regularized Partial Correlation	EBIC- glasso	Strength Betweenness Closeness	≥0.50	95% CI	Poor appetite was the central symptom.
Ma et al. [28]	Dec 2022–Apr 2023	480	53.25 ± 13.73	172 (35.80)	MHD	DSI-M	Spearman	Regularized Partial Correlation	EBIC- glasso	Bridge-Strength Bridge-Betweenness Bridge-Closeness	0.589 0.550 0.425	Not Reported	Worry and dry skin had the highest bridge strength.
Cheng et al. [4]	Mar–Apr 2025	488	63 (50, 70)	171 (35.04)	MHD	DSI-M	Spearman	Not Reported	Not reported	Strength	0.672 0.750 0.750	95% CI	Across occurrence frequency, severity, and distress, difficulty falling asleep had the highest strength and bridge strength.
T. Liu et al. [27]	Jun–Sep 2023	512	60.03 ± 13.33	208 (40.62)	MHD	DFSSBI	Spearman	Regularized Partial Correlation	EBIC- glasso	Strength Betweenness Closeness Bridge-Strength Predictability	Strength: 0.750 Bridge strength: 0.361	Not Reported	In the overall symptom-distress network, anxiety had the highest strength and bridge strength. Anxiety also had the highest strength in the frequency, severity, and distress dimensions.
Zhai et al. [21]	Feb–Jul 2023	280	53.14 ± 13.70	96 (34.30)	MHD	DSI-M	Partial Correlation	Partial Correlation	Not Reported	Strength Betweenness Closeness Bridge strength	Strength: 0.361 Bridge strength: 0.518	95% CI	Anxiety had the highest strength; fatigue had the highest bridge strength.
He et al. [26]	Jan–Apr 2023	483	58.46 ± 12.43	184 (38.10)	MHD	DSI-M	Not Reported	Regularized Partial Correlation	EBIC- glasso	Strength Betweenness Closeness Predictability	0.52	Not Reported	Sadness had the highest strength centrality.
L. Zhang, Han, et al. [22]	Mar–Jun 2024	276	57.06 ± 10.61	128 (46.38)	PD	DSI- Chinese	Not Reported	Not Reported	Not Reported	Strength Betweenness Closeness	0.514	Not Reported	The three symptoms with the highest strength were anxiety, fatigue, and difficulty falling asleep.
Zhou et al. [37]	Jan–Mar 2022	167	70.05 ± 7.40	56 (33.53)	Older Adults Receiving MHD	DSI-M	Not Reported	Not Reported	Not Reported	Strength Betweenness Closeness	≥0.50	95% CI	Nervousness and difficulty staying asleep had the highest strength.
Zheng and Wang [36]	Aug–Oct 2024	1866	Not Repo-rted	784 (42.00)	MHD	DSI-M	Not Reported	Estimate Network NIRA	Ising Sampler	Strength Expected-Influence	0.50	Not Reported	key nodes included difficulty concentrating, pruritus, anxiety, fatigue, difficulty staying asleep, and muscle soreness; sadness and anxiety were most influential in the high-burden group, versus sadness and pruritus in the low-burden group
Duan, Zhou, et al. [32]	Jun–Sep 2023	602	47.80 ± 16.80	317 (52.66)	PD	CKD- SBI	Not Reported	Not Reported	Not Reported	Strength Betweenness Closeness	0.517	95% CI	Fatigue and anxiety were the most central symptoms.
Y. Zhang et al. [35]	Aug–Oct 2023	368	54.72 ± 13.49	153 (41.58)	MHD	DSI-M	Spearman	Regularized Partial Correlation	EBIC- glasso	Strength Betweenness Closeness	0.671	Not Reported	The three symptoms with the highest strength were numbness or tingling in the feet, headache, and difficulty falling asleep.
Chang et al. [8]	Sep–Dec 2023	354	53.06 ± 10.80	147 (41.53)	MHD	DSI-M KDQ	Spearman	Regularized Partial Correlation	EBIC- glasso	Strength Betweenness Closeness Bridge strength Expected-Influence	Strength: 0.360 Bridge-Strength: 0.282 Expected-Influence: 0.360	95% CI	Worry had the highest strength and expected influence; fatigue had the highest bridge strength.
Chan et al. [31]	May–Nov 2022	268	59.87 ± 11.72	114 (42.50)	MHD	PAOFI MoCA	Spearman	Regularized Partial Correlation	EBIC- glasso Pairwise Markov Random Field	Strength	0.362	Not Reported	Difficulty solving problems and difficulty understanding instructions were the two most central complaints in the PAOFI network.
Li et al. [33]	Jul–Nov 2020	301	51.98 ± 14.31	125 (41.50)	MHD	SIS PHQ-9	Not Reported	Regularized Partial Correlation	EBIC- glasso	Strength Betweenness Closeness Bridge-strength Bridge-betweenness Bridge-closeness Predictability	Strength: 0.503 Bridge-Strength: 0.518	95% CI	In the SIS network, ‘others avoid me because of my illness’, ‘others think it is my fault,’ and ‘my job security has been affected’ were central symptoms. In the PHQ-9 network, worthlessness was central. In the combined partial-correlation network, ‘others distance themselves from me because of my illness’ had the highest strength, followed by worthlessness and perceived blame or incompetence.
Song et al. [34]	Mar–Dec 2024	558	55.45 ± 12.65	229 (41.00)	MHD	SIS DSI-M	Not Reported	Regularized Partial Correlation	EBIC- glasso	Strength Betweenness Closeness	Overall: 0.672	95% CI	Muscle soreness had the highest strength in the overall network. Chest pain and muscle soreness had the highest strength in the low-stigma group, whereas sadness had the highest strength in the moderate-stigma group.

Note. MHD, maintenance hemodialysis; PD, peritoneal dialysis; NIRA, network intervention response algorithm; EBIC, extended Bayesian information criterion; glasso, graphical least absolute shrinkage and selection operator; DSI-M, modified Dialysis Symptom Index; CKD-SBI, Chronic Kidney Disease Symptom Burden Index; KDQ, Kidney Disease Questionnaire; PAOFI, Patient Assessment of Own Functioning Inventory; MoCA, Montreal Cognitive Assessment; SIS, Social Impact Scale; PHQ-9, Patient Health Questionnaire-9; PDF, Post-Dialysis Fatigue Scale; DFSSBI, Dialysis Frequency, Severity, and Symptom Burden Index; DSI-Chinese, Chinese version of the Dialysis Symptom Index.

**Table 2:** Quality assessment scores of the included studies.

First Author	Sample and Reporting Quality				Analytic Quality										Open Science			
	Demographic Information	Clinical Information	Subgroup Description	Sample Size	Data Preprocessing		Network Analysis		Subgroup Analysis	Berkson’s Bias	Centrality Analysis	Uncertainty Assessment			Preregistration	Open Access	Open Data	Open Code
	Age, Sex, Country, etc.	Comorbidities/Symptom Duration	Definition/Sample Size	≥5–10 Observations/Node; N ≥ 100	Missing Data	Other Preprocessing	Model/Software	Selection Criteria	Methods/Comparison	Total-Score Cutoffs	Raw Values for All Indices	Centrality Stability	Edge Accuracy	Other Assessments				
Wu et al. [30]	1	1	0	1	1	1	1	0	0	1	0	1	0	0	0	0	0	0
Tian et al. [29]	1	1	0	1	0	1	1	0	0	1	0	1	1	0	0	0	0	0
Ma et al. [28]	1	1	0	1	1	1	1	0	0	1	0	1	0	0	0	0	0	0
Cheng et al. [4]	1	1	0	1	1	1	0	0	0	1	0	1	1	0	0	0	0	0
T. Liu et al. [27]	1	1	0	1	1	1	1	0	0	1	0	1	0	0	0	0	0	0
Zhai et al. [21]	1	1	0	1	1	1	1	0	0	1	0	1	1	0	0	0	0	0
He et al. [26]	1	1	0	1	1	1	1	0	0	1	0	1	0	0	0	0	0	0
L. Zhang, Han, et al. [22]	1	1	0	1	1	1	0	0	0	1	0	1	0	0	0	0	0	0
Zhou et al. [37]	1	1	0	1	1	1	0	0	0	1	0	1	1	0	0	1	0	0
Zheng and Wang [36]	1	1	1	1	1	1	1	0	1	1	0	1	0	0	0	0	0	0
Duan, Zhou, et al. [32]	1	1	0	1	1	1	0	0	0	1	0	1	1	1	0	1	0	0
Y. Zhang et al. [35]	1	1	0	1	1	1	1	0	0	1	0	1	0	0	0	1	0	0
Chang et al. [8]	1	1	0	1	1	1	1	0	0	1	0	1	1	1	0	1	0	0
Chan et al. [31]	1	1	0	1	1	1	1	1	0	1	0	1	1	0	0	1	0	0
Li et al. [33]	1	1	0	1	1	1	1	1	0	1	0	1	1	0	0	1	0	0
Song et al. [34]	1	1	1	1	1	1	1	0	1	1	0	1	1	1	0	1	0	0

## Data Availability

No new data were created or analyzed in this study.

## Supplementary Materials

The following supporting information can be downloaded at: https://www.mdpi.com/article/10.3390/healthcare14213583/s1. Supplementary Material S1: Search strategy; Table S1: Study contributions and extracted node strength ranks; Table S2: Sensitivity of centrality rankings to study selection; Table S3: Leave-one-out sensitivity analysis of centrality rankings.
