# Supplementary material for: Symptom Networks in Patients Receiving Dialysis: A Systematic Review of Node Centrality, Bridge Centrality, and Methodological Quality
Source: Healthcare (Basel). 2026 Oct 23;14(21):3583. doi: 10.3390/healthcare14213583 (PMC13763114; doi:10.3390/healthcare14213583)
Supplement: Supplementary file 1 [file healthcare-14-03583-s001.zip › healthcare-4567946-supplementary.pdf]

Supplementary Materials-S1

Search Strategy

PubMed

① ("Renal Dialysis"[Mesh] OR "Peritoneal Dialysis"[Mesh] OR "Kidney Failure, Chronic"[Mesh] OR dialys*[Title/Abstract] OR hemodialys*[Title/Abstract] OR haemodialys*[Title/Abstract] OR "peritoneal dialysis"[Title/Abstract] OR "renal replacement therap*"[Title/Abstract] OR "maintenance dialysis"[Title/Abstract] OR "maintenance hemodialysis"[Title/Abstract] OR "end stage renal disease"[Title/Abstract] OR "end-stage renal disease"[Title/Abstract] OR "end stage kidney disease"[Title/Abstract] OR "end-stage kidney disease"[Title/Abstract] OR ESRD[Title/Abstract] OR ESKD[Title/Abstract])

② ("Signs and Symptoms"[Mesh] OR symptom*[Title/Abstract] OR "symptom burden"[Title/Abstract] OR "symptom cluster*"[Title/Abstract] OR "symptom distress"[Title/Abstract] OR "patient-reported outcome*"[Title/Abstract] OR pain[Title/Abstract] OR fatigue[Title/Abstract] OR pruritus[Title/Abstract] OR itch*[Title/Abstract] OR dyspnea[Title/Abstract] OR dyspnoea[Title/Abstract] OR nausea[Title/Abstract] OR cramp*[Title/Abstract] OR dizziness[Title/Abstract] OR anorexia[Title/Abstract] OR appetite[Title/Abstract] OR sleep[Title/Abstract] OR depression[Title/Abstract] OR anxiety[Title/Abstract])

③ ("symptom network*"[Title/Abstract] OR "network analys*"[Title/Abstract] OR "network approach"[Title/Abstract] OR "network model*"[Title/Abstract] OR "network structure*"[Title/Abstract] OR "network perspective"[Title/Abstract] OR "Gaussian graphical model*"[Title/Abstract] OR GGM[Title/Abstract] OR EBICglasso[Title/Abstract] OR glasso[Title/Abstract] OR "graphical LASSO"[Title/Abstract] OR "partial correlation network*"[Title/Abstract] OR "regularized partial correlation"[Title/Abstract] OR "regularised partial correlation"[Title/Abstract] OR centrality[Title/Abstract] OR "expected influence"[Title/Abstract] OR predictability[Title/Abstract] OR "bridge symptom*"[Title/Abstract] OR "bridge centrality"[Title/Abstract] OR "cross-lagged panel network*"[Title/Abstract] OR "temporal network*"[Title/Abstract] OR "dynamic network*"[Title/Abstract])

① AND ② AND ③

Scopus

①(dialys* OR hemodialys* OR haemodialys* OR "peritoneal dialysis" OR "maintenance dialysis" OR "maintenance hemodialysis" OR "maintenance haemodialysis" OR "renal replacement therapy" OR "renal replacement therapies" OR "end stage renal disease" OR "end-stage renal disease" OR "end stage kidney disease" OR "end-stage kidney disease" OR ESRD OR ESKD)

② (symptom* OR "symptom burden" OR "symptom cluster" OR "symptom clusters" OR "symptom distress" OR pain OR fatigue OR pruritus OR itch* OR dyspnea OR dyspnoea OR nausea OR cramp* OR dizziness OR appetite OR sleep OR depression OR anxiety)

③ ("symptom network" OR "symptom networks" OR "network analysis" OR "network analyses" OR "network approach" OR "network model" OR "network models" OR "Gaussian graphical model" OR "Gaussian graphical models" OR GGM OR EBICglasso OR glasso OR "graphical LASSO" OR "partial correlation network" OR "partial correlation networks" OR centrality OR "expected influence" OR predictability OR "bridge symptom" OR "bridge symptoms" OR "bridge centrality" OR "cross-lagged panel network" OR "cross-lagged panel networks" OR "temporal network" OR "temporal networks" OR "dynamic network" OR "dynamic networks")

① AND ② AND ③

Web Of Science

① (dialys* OR hemodialys* OR haemodialys* OR "peritoneal dialysis" OR "maintenance dialysis" OR "maintenance hemodialysis" OR "renal replacement therap*" OR "end stage renal disease" OR "end-stage renal disease" OR "end stage kidney disease" OR "end-stage kidney disease" OR ESRD OR ESKD)

② (symptom* OR "symptom burden" OR "symptom cluster*" OR "symptom distress" OR "patient-reported outcome*" OR pain OR fatigue OR pruritus OR itch* OR dyspnea OR dyspnoea OR nausea OR cramp* OR dizziness OR anorexia OR appetite OR sleep OR depression OR anxiety)

③ ("symptom network*" OR "network analys*" OR "network approach" OR "network model*" OR "network structure*" OR "network perspective" OR "Gaussian graphical model*" OR GGM OR EBICglasso OR glasso OR "graphical LASSO" OR "partial correlation network*" OR "regularized partial correlation" OR "regularised partial correlation" OR centrality OR "expected influence" OR predictability OR "bridge symptom*" OR "bridge centrality" OR "cross-lagged panel network*" OR "temporal network*" OR "dynamic network*")

① AND ② AND ③

EBSCO

① (dialys* OR hemodialys* OR haemodialys* OR "peritoneal dialysis" OR "maintenance dialysis" OR "maintenance hemodialysis" OR "renal replacement therap*" OR "end stage renal disease" OR "end-stage renal disease" OR "end stage kidney disease" OR "end-stage kidney disease" OR ESRD OR ESKD)

② (symptom* OR "symptom burden" OR "symptom cluster" OR "symptom clusters" OR pain OR fatigue OR pruritus OR itch* OR dyspnea OR dyspnoea OR nausea OR cramp* OR appetite OR sleep OR depression OR anxiety)

③ ("symptom network" OR "symptom networks" OR "network analysis" OR "network analyses" OR "Gaussian graphical model" OR "Gaussian graphical models" OR GGM OR EBICglasso OR glasso OR "graphical LASSO" OR "partial correlation network" OR "partial correlation networks" OR centrality OR "expected influence" OR predictability OR "bridge centrality" OR "cross-lagged panel network" OR "cross-lagged panel networks" OR "temporal network" OR "dynamic network")

① AND ② AND ③

CNKI & WanFang
dialys* AND symptom* AND network*

Supplementary Material-S2

Table S1

Study contributions and extracted node strength ranks

Each of the nine studies contributed one network containing 18–30 symptom nodes. Thirty symptom labels were represented, with six to nine studies contributing ranking information for each symptom.

Part A Studies contributing to each symptom

Symptom	n	Contributing studies
Anxiety	9	A, B, C, D, E, F, G, H, I
Bone or joint pain	9	A, B, C, D, E, F, G, H, I
Chest pain	6	A, B, D, F, H, I
Constipation	8	A, B, C, D, F, G, H, I
Cough	8	A, B, C, D, F, G, H, I
Decreased appetite	9	A, B, C, D, E, F, G, H, I
Decreased interest in sex	7	A, B, D, E, G, H, I
Diarrhea	6	A, B, D, G, H, I
Difficulty becoming sexually aroused	6	A, B, D, E, H, I
Difficulty concentrating	6	A, B, D, G, H, I
Difficulty falling asleep	9	A, B, C, D, E, F, G, H, I
Difficulty staying asleep	9	A, B, C, D, E, F, G, H, I
Dizziness	7	A, B, D, F, G, H, I
Dry mouth	9	A, B, C, D, E, F, G, H, I
Dry skin	9	A, B, C, D, E, F, G, H, I
Fatigue	9	A, B, C, D, E, F, G, H, I
Headache	7	A, B, D, F, G, H, I
Irritability	8	A, B, D, E, F, G, H, I
Itching	9	A, B, C, D, E, F, G, H, I
Muscle cramps	9	A, B, C, D, E, F, G, H, I
Muscle soreness	8	A, B, D, E, F, G, H, I
Nausea	9	A, B, C, D, E, F, G, H, I
Nervousness	7	A, B, D, E, F, H, I
Numbness or tingling in feet	8	A, B, D, E, F, G, H, I

Symptom	n	Contributing studies
Restless legs	7	A, B, D, E, F, H, I
Sadness	9	A, B, C, D, E, F, G, H, I
Shortness of breath	8	A, B, C, D, F, G, H, I
Swelling in legs	9	A, B, C, D, E, F, G, H, I
Vomiting	9	A, B, C, D, E, F, G, H, I
Worry	9	A, B, C, D, E, F, G, H, I

Note: n counts studies with a non-missing strength rank or extraction-based rank range for the symptom. It does not count studies identifying that symptom as the most central. Missing entries were not counted. Symptoms are listed alphabetically. Study codes A–I are defined in Part C.

Part B Extracted within network strength ranks

Rank 1 indicates the highest node strength within a network. Entries are extracted ordinal ranks, not raw centrality estimates or normalized ranks.

Symptom	n	A	B	C	D	E	F	G	H	I
Anxiety	9	6	1	1	3	1	5	2	5	13
Bone or joint pain	9	20	4–5	11	25	18	18–19	6	25	27
Chest pain	6	19	24–25	—	24	—	6	—	8	3
Constipation	8	29	18–21	16	29	—	26	26	6	24
Cough	8	26	18–22	4	18	—	14	19	22	28
Decreased appetite	9	22	20–22	12	23	17	13	20	24	29
Decreased interest in sex	7	9	29–30	—	2	8	—	17	20	5
Diarrhea	6	30	28	—	26	—	—	24	10	18
Difficulty becoming sexually aroused	6	17	29–30	—	6	13	—	—	15	6
Difficulty concentrating	6	21	18–22	—	22	—	—	1	12	25
Difficulty falling asleep	9	3	4–5	5	19	3	11	9	3	15
Difficulty staying asleep	9	5	10–11	9	11	7	2	5	9	9
Dizziness	7	14	12–14	—	21	—	22	10	19	21
Dry mouth	9	27	12–14	15	14	15	23	18	14	19
Dry skin	9	4	12–14	10	8	10	15	14–15	16	16
Fatigue	9	13	9	2	5	2	20	3–4	30	30
Headache	7	18	6–8	—	15	—	21	12–13	2	4
Irritability	8	2	2	—	13	16	9–10	3–4	13	22
Itching	9	8	15–16	3	12	5	12	8	26	17
Muscle cramps	9	23	18–21	7	30	9	24	25	28	23
Muscle soreness	8	12	10–11	—	17	6	8	7	17	1
Nausea	9	7	15–16	14	7	11	4	14–15	18	20
Nervousness	7	15	6–8	—	20	21	1	—	23	14
Numbness or tingling in feet	8	25	27	—	27–28	14	16	22	1	10
Restless legs	7	24	24–25	—	27–28	19	17	—	7	26
Sadness	9	1	3	18	1	22	7	12–13	29	2
Shortness of breath	8	16	17	8	10	—	18–19	16	27	7
Swelling in legs	9	28	26	13	16	4	25	23	21	12
Vomiting	9	11	23	17	9	20	9–10	21	4	8

Symptom	n	A	B	C	D	E	F	G	H	I
Worry	9	10	6–8	6	4	12	3	11	11	11

Note: An em dash (—) indicates that the symptom was not included as a node in that analyzed network. A numerical range indicates that a unique rank could not be resolved from the published figure. These ranges represent extraction uncertainty, not confidence intervals. Each range contributes one study to n. Lower and upper bounds were normalized separately using the procedure described in Section 2.4. Study codes and network node counts are given in Part C.

Part C Study codes and extraction sources

Code	Study	Nodes	Source location
A	Wu et al. (2023)	30	p. 59, Table 2 and Figure 2 (PDF p. 4).
B	T. Liu et al. (2024)	30	pp. 2227–2228, symptom item table and the overall-network panel in Figure 3 (PDF pp. 4–5).
C	Zhai et al. (2023)	18	p. 2292, Figures 1–2 and figure legends (PDF p. 4).
D	He et al. (2025)	30	p. 613, text and Table 2; p. 614, Figure 2 (PDF pp. 2–3).
E	L. Zhang, Han, et al. (2025)	22	p. 3464, Table 1 and Figure 1; p. 3465, Figure 2 (PDF pp. 3–4).
F	Zhou et al. (2023)	26	PDF pp. 6 and 8, text; Figure 2, Degree panel (PDF p. 8), defined as strength in the methods.
G	Zheng and Wang (2025)	26	PDF p. 7, Table 2; Figure 3a, overall-network Strength panel (PDF p. 8).
H	Y. Zhang et al. (2024)	30	p. 634, Figure 1 node labels and Figure 2 (PDF p. 7).
I	Song et al. (2026)	30	PDF p. 8, Figure 3, overall-network Strength panel and legend.

Note: Nodes is the number of symptom nodes in the selected network, not the number of items in the original symptom instrument. PDF page numbers refer to the supplied full-text files. Each study contributed one network. Full bibliographic references are provided in the main manuscript.

Supplementary Material-S3

Table S2

Sensitivity of centrality rankings to study selection

Two post hoc restrictions were applied to the nine-study ranking synthesis. Sadness had the highest relative strength rank in both restricted analyses. The exact symptom ordering varied with study selection. All analyses retained the primary extraction bounds and normalization based on the number of nodes in each network.

Analysis	n	Anxiety	Difficulty falling asleep	Sadness
Primary analysis	9	0.0690	0.2353	0.2400
Exclude 18 and 22 node networks	7	0.1379	0.3200	0.0690
Restrict to 30 node networks	5	0.1379	0.1034–0.1379	0.0345

n is the number of studies. Values are median normalized ranks or bounds on those medians. Lower values indicate higher relative strength. Bounds reflect extraction uncertainty, not confidence intervals. The table displays the three leading symptoms from the primary analysis, rather than the top three symptoms in every restricted analysis.

Studies retained in each analysis

Study	Modality	Nodes	Seven-study analysis	Five-study analysis
Wu et al. (2023)	HD	30	Yes	Yes
T. Liu et al. (2024)	HD	30	Yes	Yes
Zhai et al. (2023)	HD	18	No	No
He et al. (2025)	HD	30	Yes	Yes
L. Zhang, Han, et al. (2025)	PD	22	No	No
Zhou et al. (2023)	HD	26	Yes	No
Zheng and Wang (2025)	HD	26	Yes	No
Y. Zhang et al. (2024)	HD	30	Yes	Yes
Song et al. (2026)	HD	30	Yes	Yes

HD, hemodialysis; PD, peritoneal dialysis. Zhai et al. (2023) and L. Zhang, Han, et al. (2025) retained 18 and 22 nodes after prevalence and factor-analysis selection. Both restricted subsets contain only HD studies. Equal node counts do not establish identical symptom content.

Normalization was $(r - 1)/(K - 1)$, where r is the within-network node-strength rank and K is the number of symptom nodes in that network. Missing symptoms were not assigned ranks. Each study contributed equally. Lower and upper extraction bounds were summarized separately using the median and the 25th and 75th percentiles (linear interpolation, R type 7). Restrictions changed both study populations and network composition; differences cannot be attributed solely to node selection.

Primary analysis with nine studies

Symptom-specific medians and quartiles of normalized node-strength ranks. Lower values indicate higher relative strength.

Symptom	n	Median	25th percentile	75th percentile
Anxiety	9	0.0690	0.0000	0.1600
Bone or joint pain	9	0.6800–0.7200	0.5882	0.8276
Chest pain	6	0.4310	0.2103	0.7500
Constipation	8	0.9239	0.7414–0.7672	0.9741
Cough	8	0.6531–0.7221	0.5697	0.7586
Decreased appetite	9	0.7586	0.6552–0.7241	0.7619
Decreased interest in sex	7	0.3333	0.2069	0.6476
Diarrhea	6	0.8910	0.6552	0.9283
Difficulty becoming sexually aroused	6	0.5172	0.2500	0.5665
Difficulty concentrating	6	0.6379–0.7069	0.4310–0.4569	0.7155–0.7241
Difficulty falling asleep	9	0.2353	0.0952	0.4000
Difficulty staying asleep	9	0.2759	0.1600	0.3103–0.3448
Dizziness	7	0.6207	0.4138–0.4483	0.6897
Dry mouth	9	0.6667	0.4483	0.8235
Dry skin	9	0.5172	0.3793–0.4286	0.5200–0.5294
Fatigue	9	0.2759	0.0800–0.1200	0.7600
Headache	7	0.4400–0.4800	0.1379–0.1724	0.5345
Irritability	8	0.3669–0.3869	0.0686–0.0986	0.4889
Itching	9	0.3793	0.2414	0.4828–0.5172
Muscle cramps	9	0.7586	0.5862–0.6897	0.9310
Muscle soreness	8	0.2952–0.3124	0.2395	0.4224
Nausea	9	0.4828–0.5172	0.2069	0.5862
Nervousness	7	0.4828	0.3103–0.3448	0.7069
Numbness or tingling in feet	8	0.7233	0.5276	0.8541
Restless legs	7	0.7931–0.8276	0.7166	0.8596
Sadness	9	0.2400	0.0345	0.9655
Shortness of breath	8	0.5345	0.3864	0.6200–0.6300
Swelling in legs	9	0.7059	0.5172	0.8800
Vomiting	9	0.3448–0.3600	0.2759	0.8000
Worry	9	0.3103	0.1724–0.2414	0.3448

n is the number of studies with a non-missing rank for that symptom. A range within a cell gives lower and upper extraction-based bounds on that statistic; it is not a confidence interval. The interquartile interval is defined by the 25th and 75th percentiles. Symptoms are listed alphabetically.

Restricted analysis with seven studies

Symptom-specific medians and quartiles of normalized node-strength ranks. Lower values indicate higher relative strength.

Symptom	n	Median	25th percentile	75th percentile
Anxiety	7	0.1379	0.0545	0.1662
Bone or joint pain	7	0.6800–0.7200	0.4276	0.8276
Chest pain	6	0.4310	0.2103	0.7500
Constipation	7	0.9655	0.6897–0.7414	0.9828
Cough	7	0.7200–0.7241	0.5862–0.6531	0.7931
Decreased appetite	7	0.7586	0.6897–0.7241	0.7766
Decreased interest in sex	6	0.4579	0.1724	0.6514
Diarrhea	6	0.8910	0.6552	0.9283
Difficulty becoming sexually aroused	5	0.4828	0.1724	0.5517
Difficulty concentrating	6	0.6379–0.7069	0.4310–0.4569	0.7155–0.7241
Difficulty falling asleep	7	0.3200	0.0862–0.1034	0.4414
Difficulty staying asleep	7	0.2759	0.1490	0.2931–0.3103
Dizziness	7	0.6207	0.4138–0.4483	0.6897
Dry mouth	7	0.6207	0.4483	0.7800
Dry skin	7	0.5172	0.3103–0.3448	0.5186–0.5386
Fatigue	7	0.4138	0.2069	0.8800
Headache	7	0.4400–0.4800	0.1379–0.1724	0.5345
Irritability	7	0.3200–0.3600	0.0572–0.0772	0.4138
Itching	7	0.4400	0.3297	0.5172–0.5345
Muscle cramps	7	0.9200	0.7586	0.9455
Muscle soreness	7	0.3103–0.3448	0.2600	0.4655
Nausea	7	0.4828–0.5172	0.2069	0.5531–0.5731
Nervousness	6	0.4655	0.2414–0.2931	0.6121
Numbness or tingling in feet	7	0.8276	0.4552	0.8683
Restless legs	6	0.7931–0.8103	0.6783	0.8448–0.8534
Sadness	7	0.0690	0.0172	0.3400–0.3600
Shortness of breath	7	0.5517	0.4138	0.6400–0.6600
Swelling in legs	7	0.8621	0.6034	0.9055
Vomiting	7	0.3200–0.3448	0.2586	0.5517–0.5593
Worry	7	0.3103	0.1379–0.1724	0.3448

n is the number of studies with a non-missing rank for that symptom. A range within a cell gives lower and upper extraction-based bounds on that statistic; it is not a confidence interval. The interquartile interval is defined by the 25th and 75th percentiles. Symptoms are listed alphabetically.

Restricted analysis with five studies

Symptom-specific medians and quartiles of normalized node-strength ranks. Lower values indicate higher relative strength.

Symptom	n	Median	25th percentile	75th percentile
Anxiety	5	0.1379	0.0690	0.1724
Bone or joint pain	5	0.8276	0.6552	0.8276
Chest pain	5	0.6207	0.2414	0.7931
Constipation	5	0.7931	0.5862–0.6897	0.9655
Cough	5	0.7241	0.5862–0.7241	0.8621
Decreased appetite	5	0.7586	0.7241	0.7931
Decreased interest in sex	5	0.2759	0.1379	0.6552
Diarrhea	5	0.8621	0.5862	0.9310
Difficulty becoming sexually aroused	5	0.4828	0.1724	0.5517
Difficulty concentrating	5	0.6897–0.7241	0.5862–0.6897	0.7241
Difficulty falling asleep	5	0.1034–0.1379	0.0690	0.4828
Difficulty staying asleep	5	0.2759	0.2759	0.3103–0.3448
Dizziness	5	0.6207	0.4483	0.6897
Dry mouth	5	0.4483	0.4483	0.6207
Dry skin	5	0.3793–0.4483	0.2414	0.5172
Fatigue	5	0.4138	0.2759	1.0000
Headache	5	0.1724–0.2414	0.1034	0.4828
Irritability	5	0.4138	0.0345	0.4138
Itching	5	0.4828–0.5172	0.3793	0.5517
Muscle cramps	5	0.7586	0.7586	0.9310
Muscle soreness	5	0.3793	0.3103–0.3448	0.5517
Nausea	5	0.4828–0.5172	0.2069	0.5862
Nervousness	5	0.4828	0.4483	0.6552
Numbness or tingling in feet	5	0.8276	0.3103	0.8966
Restless legs	5	0.7931–0.8276	0.7931	0.8621
Sadness	5	0.0345	0.0000	0.0690
Shortness of breath	5	0.5172	0.3103	0.5517
Swelling in legs	5	0.6897	0.5172	0.8621
Vomiting	5	0.2759	0.2414	0.3448
Worry	5	0.3103	0.1724–0.2414	0.3448

n is the number of studies with a non-missing rank for that symptom. A range within a cell gives lower and upper extraction-based bounds on that statistic; it is not a confidence interval. The interquartile interval is defined by the 25th and 75th percentiles. Symptoms are listed alphabetically.

Supplementary Material-S4

Table S3

Leave one out sensitivity analysis of centrality rankings

Each of the nine studies was omitted in turn, and the synthesis was repeated with the remaining eight studies. Anxiety retained the highest relative strength rank in every iteration. The relative positions of difficulty falling asleep and sadness varied across omissions.

Study omitted	Anxiety	Difficulty falling asleep	Sadness
Wu et al. (2023)	0.0545	0.2776	0.3400–0.3600
T. Liu et al. (2024)	0.1034	0.2776	0.3400–0.3600
Zhai et al. (2023)	0.1034	0.2117–0.2290	0.1545
He et al. (2025)	0.0890	0.1694–0.1866	0.3400–0.3600
L. Zhang, Han, et al. (2025)	0.1034	0.2776	0.1545
Zhou et al. (2023)	0.0545	0.1694–0.1866	0.2545–0.2745
Zheng and Wang (2025)	0.1034	0.1694–0.1866	0.1545
Y. Zhang et al. (2024)	0.0545	0.2776	0.1545
Song et al. (2026)	0.0545	0.1694–0.1866	0.3400–0.3600

Values are medians of normalized node-strength ranks, with extraction-based bounds where a unique median could not be established. Each displayed symptom was represented in all eight retained studies in every iteration. Lower values indicate higher relative strength.

All iterations used the same symptom definitions, within-network normalization $(r - 1)/(K - 1)$, missing-value handling and equal study weights as the primary analysis. Lower and upper extraction bounds were propagated separately. These bounds describe uncertainty in extracting ranks from published figures and are not confidence intervals.

The table focuses on the three leading symptoms in the primary synthesis. Their primary median normalized ranks were 0.0690 for anxiety, 0.2353 for difficulty falling asleep and 0.2400 for sadness. A high position in these descriptive syntheses does not establish equal absolute centrality across networks.

Variation across all nine omissions

For each symptom, the table gives the range of contributing-study counts and the smallest lower bound and largest upper bound of the median across the nine leave-one-out iterations.

Symptom	Contributing studies	Minimum median bound	Maximum median bound
Anxiety	8	0.0545	0.1034
Bone or joint pain	8	0.6676	0.7648
Chest pain	5–6	0.2414	0.6207
Constipation	7–8	0.8824	0.9655
Cough	7–8	0.5862	0.7241
Decreased appetite	8	0.7414	0.7593
Decreased interest in sex	6–7	0.3046	0.4867
Diarrhea	5–6	0.8621	0.9200
Difficulty becoming sexually aroused	5–6	0.4828	0.5517
Difficulty concentrating	5–6	0.5862	0.7241
Difficulty falling asleep	8	0.1694	0.2776
Difficulty staying asleep	8	0.2759	0.2808
Dizziness	6–7	0.5345	0.6552
Dry mouth	8	0.6437	0.6733
Dry skin	8	0.4729	0.5172
Fatigue	8	0.2069	0.3448
Headache	6–7	0.3062	0.4814
Irritability	7–8	0.3200	0.4138
Itching	8	0.3297	0.4097
Muscle cramps	8	0.7586	0.8393
Muscle soreness	7–8	0.2800	0.3448
Nausea	8	0.4795	0.5386
Nervousness	6–7	0.4655	0.5690
Numbness or tingling in feet	7–8	0.6190	0.8276
Restless legs	6–7	0.7931	0.8424
Sadness	8	0.1545	0.3600
Shortness of breath	7–8	0.5172	0.5517
Swelling in legs	8	0.6978	0.7840
Vomiting	8	0.3324	0.5593

Symptom	Contributing studies	Minimum median bound	Maximum median bound
Worry	8	0.3022	0.3276

The two median-bound columns jointly reflect variation across study omissions and retained extraction uncertainty. They are descriptive extrema, not confidence intervals or exact rank ranges.
